# Transmission Electron Microscopy Peeled Surface Defect of Perovskite Quantum Dots to Improve Crystal Structure

**DOI:** 10.3390/ma16176010

**Published:** 2023-09-01

**Authors:** Longfei Yuan, Taixin Zhou, Fengmin Jin, Guohong Liang, Yuxiang Liao, Aijuan Zhao, Wenbo Yan

**Affiliations:** 1 School of Chemical Engineering and Technology, Tianjin University, Tianjin 300350, China; yuanlongfei@tju.edu.cn (L.Y.); 3021207102@tju.edu.cn (T.Z.); fmjin@tju.edu.cn (F.J.); 3021207244@tju.edu.cn (Y.L.); zhaoaijuan@tju.edu.cn (A.Z.); 2 State Key Laboratory of Reliability and Intelligence of Electrical Equipment, School of Materials Science and Engineering, Hebei University of Technology, Tianjin 300130, China; yanwenbo@hebut.edu.cn

**Keywords:** transmission electron microscopy, perovskite quantum dots, [PbBr_6_]^4−^ octahedron, defect

## Abstract

Transmission electron microscopy (TEM) is an excellent characterization method to analyze the size, morphology, crystalline state, and microstructure of perovskite quantum dots (PeQDs). Nevertheless, the electron beam of TEM as an illumination source provides high energy, which causes morphological variation (fusion and melting) and recession of the crystalline structure in low radiolysis tolerance specimens. Hence, a novel and facile strategy is proposed: electron beam peel [PbBr_6_]^4−^ octahedron defects from the surface of QDs to optimize the crystal structure. TEM and high-angle annular dark-field scanning TEM (HAADF) tests indicate that the [PbBr_6_]^4−^ octahedron would be peeled from the surface of QDs when QDs samples were irradiated under high-power irradiation, and then a clear image would be obtained. To avoid interference from a protective film of “carbon deposits” on the surface of the sample when using high resolution TEM, amorphous carbon film (15–20 nm) was deposited on the surface of QDs film and then characterized by TEM and HAADF. The detection consequences showed that the defection of PbBr_2_ on the surface of QDs will gradually disappear with the extension of radiation time, which further verifies the conjecture.

## 1. Introduction

Metal halide perovskite quantum dots (PeQDs) have come into public view as superior semiconductor luminescent materials owing to their adjustable band gap, excellent electroluminescence, high charge mobility, long carrier lifetime, and high photoluminescence quantum yield [1,2,3,4,5,6]. In addition, because of their excellent properties, perovskites have shown great prospects in optoelectronic fields and have been successfully used in photovoltaic cells, photodetectors, lasers, light-emitting diodes, and many other fields [7,8,9,10,11]. In order to achieve both intuitive and convenient detection of QDs, especially in terms of their size, morphology, crystalline state, and microstructure, an excellent characterization method is urgently needed. Transmission electron microscopy (TEM) with high resolution and magnification is one of the most crucial tools for crystal structure and property characterizations of QDs [12,13]. However, the electron beam of TEM as an illumination source provides high energy, which causes morphological variation (fusion and melting) and recession of the crystalline structure in low radiolysis tolerance specimens [14,15,16]. PeQDs, a typical electron beam-sensitive material exposed to hundreds to thousands of electrons, would break down after being focused onto the microscopy and then arise radiation defects [17]. At present, the effect of low-energy electrons on the crystal surface properties has been reported, but the influence of electron beams on the crystal structure of PeQDs has not been studied [18,19]. As a result, the quality and reliability of reported imaging results are frequently questioned. It’s practical significance to achieve lower-damaged characterization of morphology, particle size, and crystalline state of PeQDs.

In response to the phenomenon of electron beam irradiation degradation of CsPbX_3_, researchers use different methods to increase imaging resolution, such as low-voltage high-resolution electron microscopy (LVHREM), cryo-TEM, carbon sedimentation, and so on [20,21,22,23]. For example, by reducing the irradiation dose rate combined with outgoing wave reconstruction, Yu revealed the microstructure of CsPbBr_3_ using spherical aberration-corrected high-resolution transmission electron microscopy (AC-HRTEM) with an accelerating voltage of 80 kV [24]. Dos Reis confirmed the tetragonal phase structure of CsPbBr_3_ PeQDs in terms of their diffraction pattern and higher-order Laue zone symmetry using converging beam electron diffraction (CBED). Furthermore, Dos Reis obtained an image of Br under low dose conditions combined with electron stack diffraction imaging, which provided a new solution for imaging PeQDs materials [25]. Li acquired diffraction images of the atomic structure of MAPbI_3_ utilizing cryo-TEM and quantified its cryocritical dose to 12 e^−^/Å^2^ at a spatial resolution of 1.49 Å [26]. Cai successfully made an amorphous carbon film with a thickness of about 10 nm deposited on the cross-sectional sample of the perovskite device [27]. After that, perovskites were irradiated under scanning transmission electron microscopy (STEM) for a period of time without any damage, indicating that the amorphous carbon film can effectively protect the perovskite layer. Due to the small size of PeQDs down to the nanometer level, incomplete coordination of atoms would occur on the QD surface, which would form some surface defects to capture excitons, reducing photoluminescence quantum yield and making it difficult to obtain complete crystal structure information of QDs [28,29,30]. Therefore, most researchers have proven modification strategies such as halide ion pairs and inorganic ligands to passivate the surface defects for improving and optimizing their crystal structure and the luminous efficiency of PeQDs [31,32].

In this study, a simple and reliable TEM approach was developed to utilize a high-energy electron beam to preprocess the PeQDs sample for peeling [PbBr6]^4−^ octahedron defects from the QDs surface and then obtaining high-resolution images. Firstly, PeQDs were prepared by means of the hot injection method, then using photoluminescence (PL) and ultraviolet-visible absorption spectra (UV-vis) to characterize the optical properties as well as an X-ray diffractometer (XRD) to characterize the crystal structure. Then the perovskite sample was irradiated under the electron beam of TEM. TEM and high-angle annular dark-field scanning TEM (HAADF) tests indicate that the [PbBr_6_]^4−^ octahedron defect of the PeQDs surface would be peeled from the surface of the QDs when irradiated at high magnification, ultimately resulting in clear images as the duration of irradiation is prolonged. To avoid interference from a protective film of “carbon deposits” on the surface of the sample irradiated under HR-TEM, the amorphous carbon film (15–20 nm) was deposited on the surface of the perovskite sample and then characterized by TEM and HAADF. Our results finally showed that the defection of PbBr_2_ on the surface of QDs will gradually disappear with the extension of radiation time, which further verified the conjecture.

## 2. Materials and Methods

### 2.1. Materials

Cs_2_CO_3_ (99.9%), oleic acid (OA, 85%), oleylamine (OAm, 80–90%), PbBr_2_ (99.999%), n-hexane (97.0%), 1-octadecene (ODE, 90%), and methyl acetate (99%) were purchased from Aladdin (Shanghai, China). All materials are used directly without reprocessing.

### 2.2. Preparation of CsPbBr_3_ QDs

Synthesis of Cs-precursors: 0.20 g of Cs_2_CO_3_, 10 mL of octadecene (ODE), and 1 mL of OA were added to a 3-neck flask. A vacuum pump was used to remove water and oxygen for 15 min at room temperature. Then the temperature was increased to 120 °C and dried for another 15 min. And the system was replaced with argon for inert gas protection, and the temperature was lowered to 90 °C for use.

Preparation of CsPbBr_3_ QDs: 102.7 mg of PbBr_2_, 7.5 mL of ODE, 1 mL of OA, and 1 mL of OAm were added to a 3-neck flask. A vacuum pump was used to remove water and oxygen for 15 min at room temperature. Then the temperature was increased to 120 °C and dried for another 15 min. And the system was replaced with argon for inert gas protection, and the temperature was raised to 160 °C. An amount of 0.8 mL of Cs-precursors was quickly injected after the temperature stabilized. The solution changed from colorless to yellow after a 10 s reaction under violent agitation. Then, the reaction was terminated by cooling in an ice-water bath.

Purification of CsPbBr_3_ QDs: The crude product was centrifuged for 5 min at 9500 rpm/min to remove salts that were not involved in the reaction. After removing the supernatant, the precipitate was dispersed into 10 mL of n-hexane. The CsPbBr_3_ PeQDs were centrifuged for 5 min at 9500 rpm/min to remove large particles, and then the supernatant was collected for testing.

### 2.3. Characterization of CsPbBr_3_ QDs

The morphology and structure of CsPbBr_3_ QDs were characterized by TEM (JEM-F200, JEOL, Tokyo, Japan) with a 200 kV electron beam accelerating voltage.

TEM observation: The condenser lens aperture was at No. 1 (aperture diameter 200 µm), the spot size was at No. 1 (spot diameter about 1 nm), and the convergence angle was about 75.6 mrad. The exposure time for TEM image acquisition was 0.5 s. The current density of the electron beam was about 17 pA/cm^2^.

STEM and HAADF observation: The condenser lens aperture was at No. 3 (aperture diameter 40 µm), the probe size was at No. 5 (probe diameter about 0.26 nm), and the camera length was at 120 mm. The probe convergence angle was about 20 mrad, and the angular range of the STEM BF detector was 25.2 mrad; the angular range of the HAADF detector was from 62.8 to 230 mrad. The dwell time of each pixel during HAADF image acquisition was 80 μs, and the size of all STEM BF and HAADF images in this work was 512 × 512 pixel^2^.

### 2.4. Carbon Coating

The CsPbBr_3_ QDs specimens were transferred to a high-vacuum sputter coater for protecting the layer deposition (JEE-4X, JEOL, Tokyo, Japan). Amorphous carbon layers with a thickness of 15–20 nm were coated on QDs specimens using pulsed carbon evaporation at 5 × 10^−6^ pa, where the sputtering current is above 50 A.

## 3. Results and Discussion

**Figure 1:**
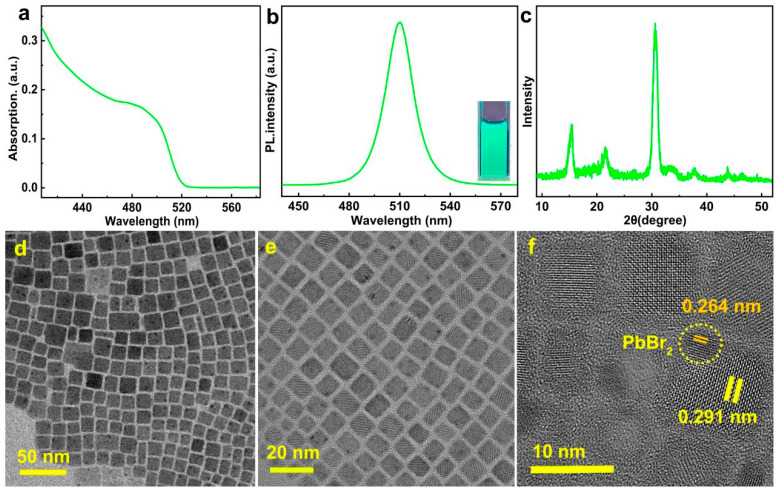
(**a**) UV-visible absorption spectroscopy; (**b**) PL spectroscopy (inset is a luminescence photograph under a 365 nm UV lamp); (**c**) X-ray diffraction; (**d**,**e**) TEM pictures in different multiples; (**f**) HRTEM of CsPbBr_3_ QDs.

The green-emission QDs were prepared through the hot-injection method using organic long-chain oleic acid (OA) and oleylamine (OAm) as ligands. As shown in Figure 1, ultraviolet visible (UV-vis) absorption and photoluminescence (PL) were carried out to explore their optical properties. In Figure 1a, UV-vis absorption spectra show that CsPbBr_3_ QDs have a distinct absorption peak at 485 nm. The steady-state PL image shows that the emission peak of CsPbBr_3_ QDs is at 510 nm and emits bright green light under 365 nm UV irradiation. It can be proven from the excitation spectrum of the 510 nm band (Figure S1) that the best excitation wavelength is 365 nm. Furthermore, the half-peak width of the QD emission peak was calculated at 19 nm, indicating that the particle size of CsPbBr_3_ QDs distributed uniformly. The result of X-ray diffraction (XRD) (Figure 1c) showed that three strong diffraction peaks at 14.9°, 21.0°, and 29.9° correspond to the (100), (110) and (200) crystal planes, respectively. The high intensity and sharp peak of the diffraction peaks indicate QDs with good crystal structure.

In order to further investigate the size and morphology of CsPbBr_3_ QDs, TEM testing was performed (Figure 1d). From the TEM morphology result of CsPbBr_3_ QDs, it can be seen that the grain size of the QDs is uniform, mostly within the range of 7–11 nm. To provide a more accurate description, 50 randomly selected grains were analyzed for particle size distribution (Figure S2). Over 80% of particles showed a particle size between 7 and 11 nm, with the smallest grain size at around 7 nm and the largest at around 12 nm, and few particle sizes are out of the range 7–11 nm. In Figure 1d,e, it is indicated that there are many “black spots” on the surface of CsPbBr_3_ QDs measured at about 2 nm. According to our research, these “black spots” are the surface defects of the QDs. Due to the defects were presence on the surface of QDs, this resulted in a low photoluminescence yield (PLQY) of 42.38% (Table S1) for CsPbBr_3_ QDs. Time-resolved PL (TRPL) was used to further test the lifetime of CsPbBr_3_ QDs and perform fitting. The TRPL spectrum (Figure S3) shows that the nonradiative recombination lifetime *τ_2_* caused by defects is 5.86 ns, accounting for 51.15%, which indicates that there are many defects on the surface of QDs. High-resolution TEM (HRTEM) testing was performed to further investigate the structure of “black spots”. As shown in Figure 1f, the interplanar spacing of “black spots” is 0.264 nm, corresponding to the (031) plane of the [PbBr_6_]^4−^ octahedron, which indicates the “black spots” formed by the surface uncoordinated [PbBr_6_]^4−^ octahedron. The interplanar spacing of 0.291 nm corresponds to the (200) plane of CsPbBr_3_ QDs.

**Figure 2:**
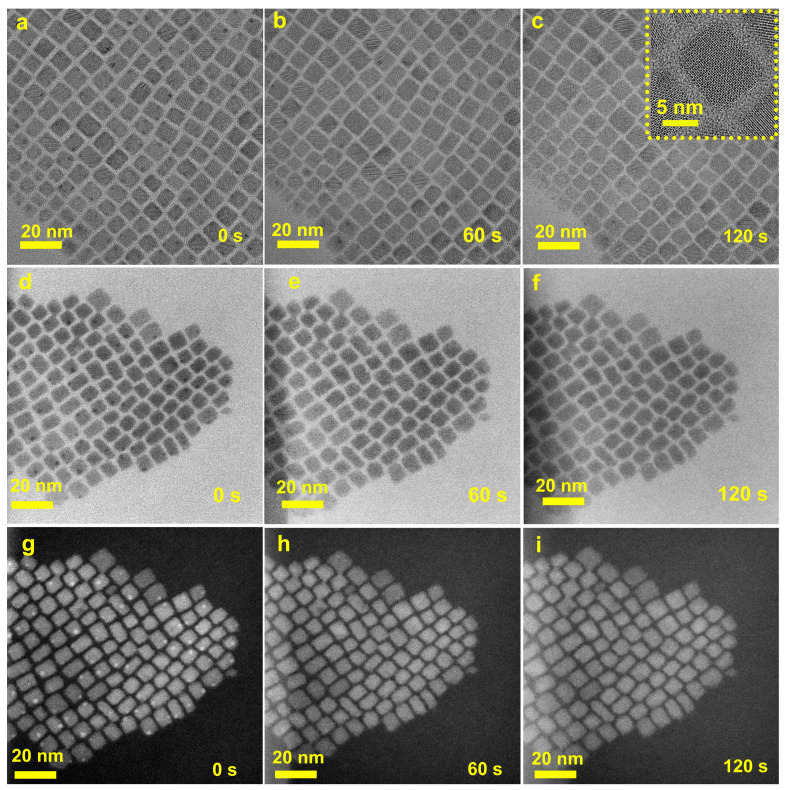
(**a**–**c**) TEM (*the insets in (**c**) is the HRTEM image of CsPbBr_3_ QDs*), STEM BF (**d**–**f**), and HAADF (**g**–**i**) images of CsPbBr_3_ QDs after exposure to electron beam irradiation for varying durations.

When we tried to adjust the magnification of the TEM to 200 k, a morphology image of CsPbBr_3_ QDs was immediately taken, as shown in Figure 2a. There are many “black spots” on the surface of CsPbBr_3_ QDs formed by [PbBr_6_]^4−^ octahedron. After radiation for 60 s at this magnification, the morphology image was obtained again (Figure 2b). The TEM result indicated that “black spots” on the surface of QDs were significantly reduced. When the radiation time reached 120 s (Figure 2c), the “black spots” were rarely observed. In order to better present the change process, corresponding recordings were made using screen recording. As observed in the Video S1, the “black spots” on the QD’s surface were very obvious at the beginning of shooting, but after high magnification radiation, the “black spots” slowly decreased until they disappeared completely. It can be attributed to the fact that the electron beam (17 pA/cm^2^) peeled [PbBr_6_]^4−^ octahedron from the surface of the QDs to reduce surface defects, resulting in a decrease in “dark spots” on the QDs surface. In order to further verify the hypothesis, scanning transmission electron microscopy bright field (STEM BF) and HAADF tests were performed on the QDs (0.16 nA, camera length 120 mm, probe size 5). Due to the fact that [PbBr_6_]^4−^ is a heavy element, it appears as “black spots” in STEM BF and “bright spots” under HAADF test conditions. It can be seen from Figure 2d,g that there are many “black spots” or “bright spots” on the surface of the QDs caused by [PbBr_6_]^4−^ defects. STEM BF and HAADF test results show that as radiation time increases, the “black spots” or “bright spots” gradually decrease (Figure 2e,f,h,i). When the radiation time increases to 120 s, the “black spots” or “bright spots” basically disappear, which is consistent with TEM results (Figure 2).

**Figure 3:**
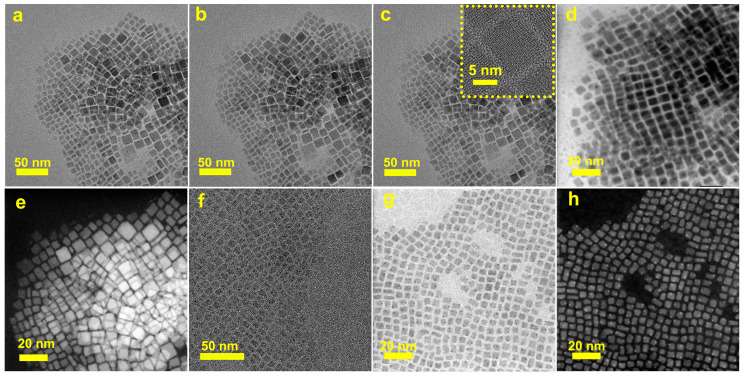
(**a**–**c**) The TEM images of CsPbBr_3_ QDs after exposure to electron beam irradiation for varying durations (*the insets in (**c**) is the HRTEM image of CsPbBr_3_ QDs*); (**d**,**e**) respectively show the STEM BF and HAADF images of the sample in figure (**a**); the TEM (**f**), STEM BF (**g**), and HAADF (**h**) CsPbBr_3_ QDs were deposited with a layer of amorphous carbon.

**Figure 4:**
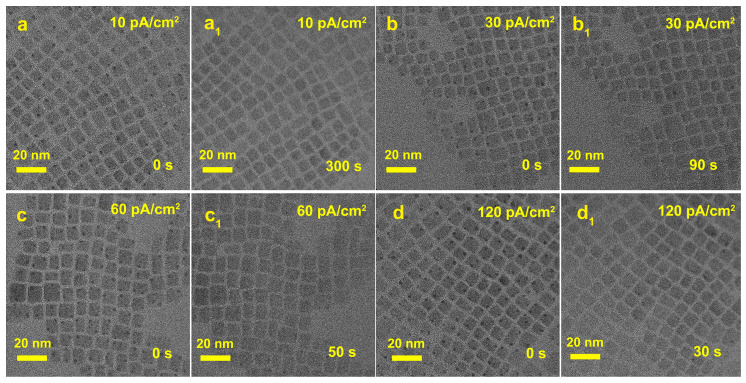
TEM images of CsPbBr_3_ QDs after exposure to electron beam irradiation for varying durations with different beam current densities (**a**,**a_1_**) 10 pA/cm^2^, (**b**,**b_1_**) 30 pA/cm^2^, (**c**,**c_1_**) 60 pA/cm^2^, (**d**,**d_1_**) 120 pA/cm^2^.

As shown in Figure 3a–c, a sample region was first selected and subjected to electron beam irradiation for varying durations. Under TEM mode, the defects on the surface of QDs were stripped away, resulting in an improvement in the crystal structure. The sample was subsequently switched to STEM mode for testing. The STEM BF (Figure 3d) and HAADF (Figure 3e) results indicated that there were indeed no under-coordinated [PbBr_6_]^4−^ deep-level defects on the QDs surface. Furthermore, it is also demonstrated the ability of electron beams to peel away surface defects and obtain perovskites with better crystal structures for clear, high-resolution images. Under conditions of high magnification of the tests, the irradiation of the QDs by an electron beam could cause organic small-molecule ligands to form a layer of “carbonaceous residue” on the sample surface, which may have covered up surface defects and consequently affected sample observation. In order to avoid interference from this carbonaceous residue, a method of treating the perovskite sample with a surface deposition of an amorphous carbon film (15–20 nm) was used before TEM and HAADF characterization. Note that the defects are not covered up by the layer of amorphous carbon film deposited on the QDs surface, as we can clearly observe the defects on the surface of the QDs from the test results (Figure 3f–h) caused by under-coordinated [PbBr_6_]^4−^. It was further confirmed that the disappearance of defects is attributed to the stripping effect of the electron beam. In order to elaborate on the underlying mechanisms causing the observed morphological variations, the difference current density of the electron beam was used to obtain TEM images of QDs. As shown in Figure 4, the time for [PbBr_6_]^4−^ octahedron defects to be completely peeled from the QD’s surface becomes shorter (from 300 s to 30 s) with the electron beam current density increasing from 10 to 120 pA/cm^2^. It indicated that the coordination interaction with Cs and [PbBr_6_]^4−^ octahedron was more easily dissociated when the energy of the electron beam was increased.

## 4. Conclusions

In this work, a novel method was proposed using electron beam irradiation to remove surface defects from QDs and obtain clear, high-resolution images. Typically, when QDs are synthesized by the method of hot injection, uncoordinated Pb vacancy defects will form on the surface, which can affect the analysis of the QDs crystal structure. TEM and HAADF results indicated that the [PbBr_6_]^4−^ octahedron could be removed from the surface of the QDs when the beam energy is stronger than the binding energy between Cs and [PbBr_6_]^4−^. Thereby, complete crystal information for CsPbBr_3_ QDs was obtained by avoiding the interference of defects in the crystal structure. This method provides a new approach to preprocess sensitive crystalline materials and analyze their atomic-scale structure and chemical properties by using the irradiation energy of electron beams.

## Data Availability

All data included in this study are available upon request by contacting the corresponding authors.

## Supplementary Materials

The following supporting information can be downloaded at: https://www.mdpi.com/article/10.3390/ma16176010/s1. Figure S1: The excitation spectrum of the 510 nm band; Figure S2: The grain size of 50 CsPbBr_3_ QDs grains were collected for each group; Figure S3: The TRPL of CsPbBr_3_ QDs; Table S1: Optical parameters of CsPbBr_3_ QDs.
