# Supplementary material for: Transmission Electron Microscopy Peeled Surface Defect of Perovskite Quantum Dots to Improve Crystal Structure
Source: Materials (Basel). 2023 Sep 1;16(17):6010. doi: 10.3390/ma16176010 (PMC10489022; doi:10.3390/ma16176010)
Supplement: Supplementary file 1 [file materials-16-06010-s001.zip › materials-2469565-supplementary.pdf]

Transmission Electron Microscopy Peeled Surface Defect of Perovskite Quantum Dots to Improve Crystal Structure

Longfei Yuan [1,†], Taixin Zhou [1,†], Fengmin Jin [1], Guohong Liang [1,*], Yuxiang Liao [1], Aijuan Zhao [1] and Wenbo Yan [2]

[1] School of Chemical Engineering and Technology, Tianjin University, Tianjin 300350, China

[2] State Key Laboratory of Reliability and Intelligence of Electrical Equipment, School of Materials Science and Engineering, Hebei University of Technology, Tianjin 300130, China

* Correspondence: guohong.liang@tju.edu.cn

† These authors contributed equally to this work.

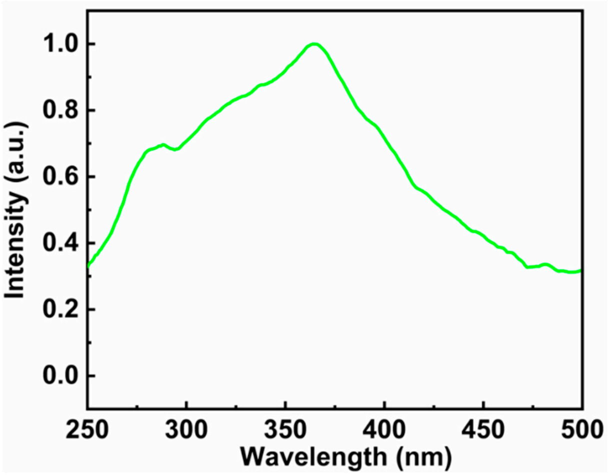

Figure S1. The excitation spectrum of the 510 nm band.

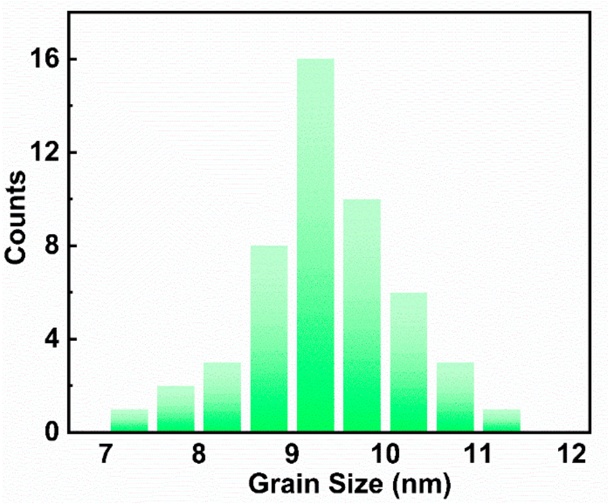

Figure S2. The grain size of 50 $CsPbBr_3$ QDs grains were collected for each group.

Table S1. Optical parameters of $CsPbBr_3$ QDs.

	λ_{em} (nm)	FWHM (nm)	PLQY (%)	τ_1 (ns)	f_1 (%)	τ_2 (ns)	f_2 (%)	τ_{ave} (ns)
$CsPbBr_3$	510	19	42.38	7.2	25.6	1.9	74.4	2.4

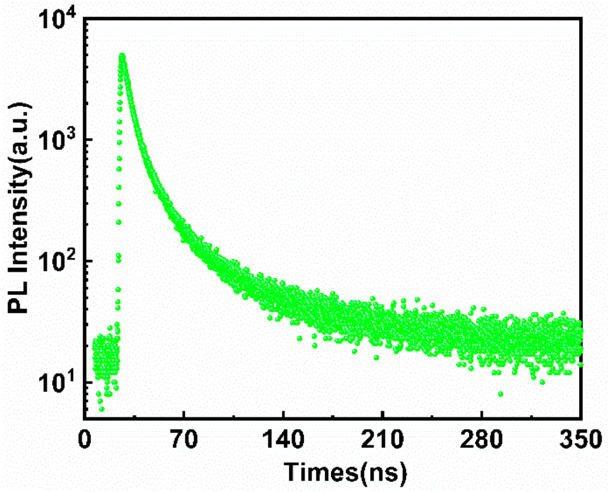

Figure S3. The TRPL of $CsPbBr_3$ QDs.
